# Pacemaker Lead Endocarditis Secondary to Staphylococcus aureus Infection From a Flash Glucose Monitor Insertion Site in a Patient With Type 1 Diabetes: A Case Report

**DOI:** 10.7759/cureus.108483

**Published:** 2026-05-08

**Authors:** Kyaw Linn, Hamza Al-Hayek, Zahra Parsian, Sanjalee Dalpathadu

**Affiliations:** 1 Endocrinology and Diabetes, York and Scarborough Teaching Hospitals NHS Foundation Trust, York, GBR; 2 Internal Medicine, York and Scarborough Teaching Hospitals NHS Foundation Trust, York, GBR; 3 General Practice, York and Scarborough Teaching Hospitals NHS Foundation Trust, York, GBR; 4 Foundation Training, York and Scarborough Teaching Hospitals NHS Foundation Trust, York, GBR

**Keywords:** device-associated endocarditis, flash glucose monitoring, flash glucose monitoring device complication, glenohumeral joint infection, glucose monitoring device complications, pacemaker endocarditis, pacemaker lead vegetation, staphylococcus aureus bacteremia, staphylococcus aureus endocarditis

## Abstract

Flash glucose monitoring (FGM) systems are widely used in diabetes care, although cutaneous complications remain uncommon. However, FGM insertion-site infections can serve as a portal for *Staphylococcus aureus*, potentially leading to systemic complications in high-risk patients. We report the case of a 46-year-old man with poorly controlled type 1 diabetes who presented with fever, confusion, and cellulitis at an FGM site. Cultures confirmed persistent *S. aureus* bacteremia. Imaging revealed deep glenohumeral joint infection requiring surgical washout. Despite an initially normal transthoracic echocardiogram, persistent bacteremia prompted transesophageal echocardiography, which demonstrated a pacemaker lead vegetation. He underwent complete device extraction and received prolonged IV flucloxacillin. This case highlights the potential for FGM-site infections to progress to septic arthritis and pacemaker lead infective endocarditis, emphasizing early recognition and aggressive management.

## Introduction

Flash glucose monitoring (FGM) and continuous glucose monitoring (CGM) systems have transformed diabetes management by providing real-time glucose trends and reducing the limitations of traditional capillary monitoring [1-4]. Their use has expanded, particularly in individuals with type 1 diabetes [5]. Although generally safe, CGM/FGM devices may cause cutaneous complications ranging from mild irritation to cellulitis, abscess formation, and, in rare cases, severe soft tissue infection [6,7]. Systematic reviews report low but clinically relevant infection rates [6]. Poor glycemic control further increases susceptibility to infection [2,3].

*Staphylococcus aureus* is a leading cause of skin and soft tissue infection and carries a high risk of bacteremia. In patients with cardiac implantable electronic devices, persistent* S. aureus *bacteremia warrants prompt investigation for device-related infective endocarditis [8]. We describe a rare progression from an FGM insertion-site infection to persistent bacteremia, septic arthritis, and pacemaker lead endocarditis.

## Case presentation

A 46-year-old man presented with a two-day history of fever, confusion, and worsening pain in the left upper arm. His medical history included type 1 diabetes mellitus (HbA1c 78 mmol/mol), hypertension, prior ischemic stroke, a permanent dual-chamber pacemaker implanted six years prior for complete heart block, and a recent nonoperative left proximal humeral fracture.

His FGM insertion site showed extensive erythema, ulceration, and purulent discharge, with tenderness extending to the shoulder (Figure 1).

**Figure 1:**
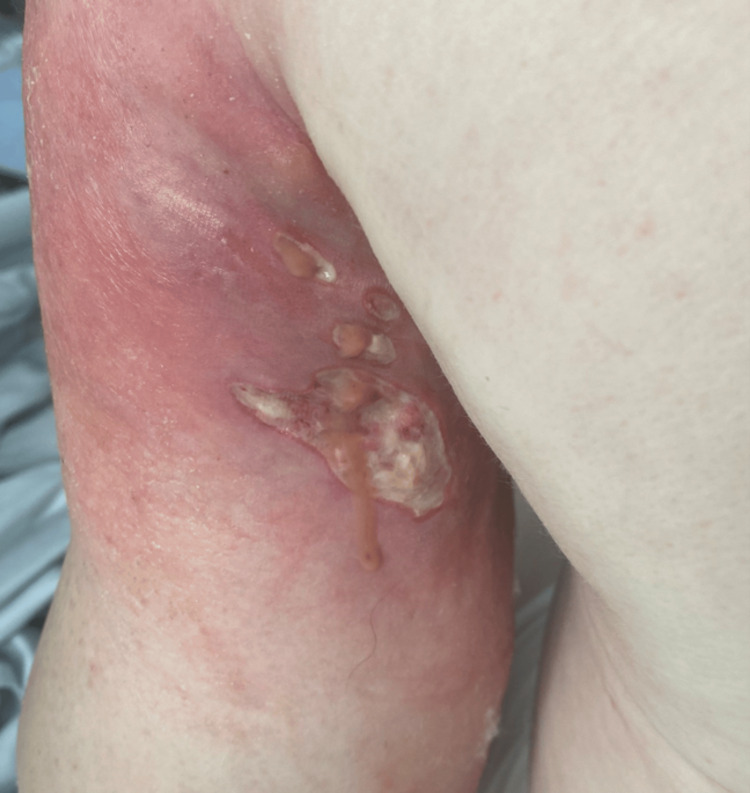
Ulcerated, purulent, and erythematous lesion at the flash glucose sensor insertion site on admission

His blood tests, as seen in Table 1, were consistent with sepsis and mild diabetic ketoacidosis. His initial blood cultures on the day of admission (Day 0) grew* S. aureus *in all four bottles. Despite switching from piperacillin-tazobactam to IV flucloxacillin, repeat cultures on Days 2, 4, and 6 remained positive. The first negative blood culture was obtained on Day 10.

**Table 1:** Blood results at admission Raised CRP is suggestive of acute infection.

Test	Result	Normal range	Units
WBC	9.8	4.0-11.0	× 10⁹/L
CRP	421	<5	mg/L
pH	7.3	7.35-7.45	-
Lactate	3.5	0.6-2.5	mmol/L
Glucose	>24	3.6-5.6	mmol/L
Ketones	2	<0.6	mmol/L

Shoulder X-ray (Figure 2) demonstrated intra-articular gas. A PET-CT scan revealed a large glenohumeral joint effusion with multifocal FDG uptake (Figure 3), consistent with septic arthritis and deep soft tissue abscesses. A surgical washout was performed on Day 27, and cultures taken on this day grew *S. aureus*.

**Figure 2:**
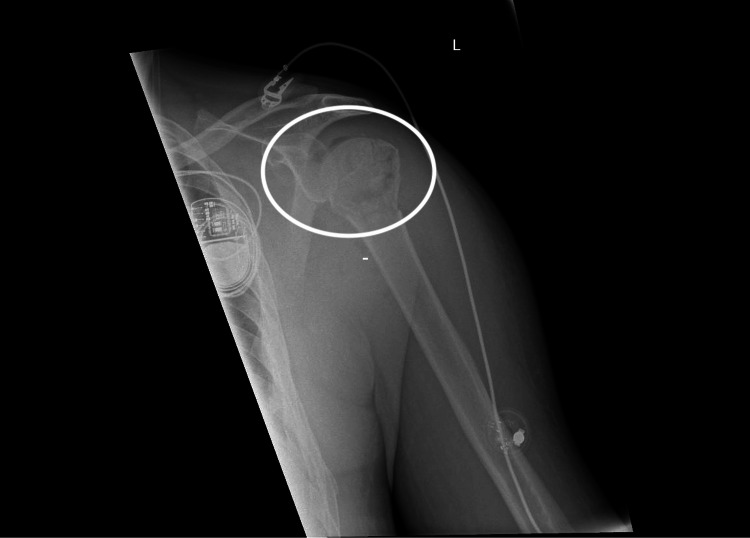
Left shoulder X-ray showing intra-articular gas In the context of the clinical presentation, this is suggestive of septic arthritis of the shoulder joint.

**Figure 3:**
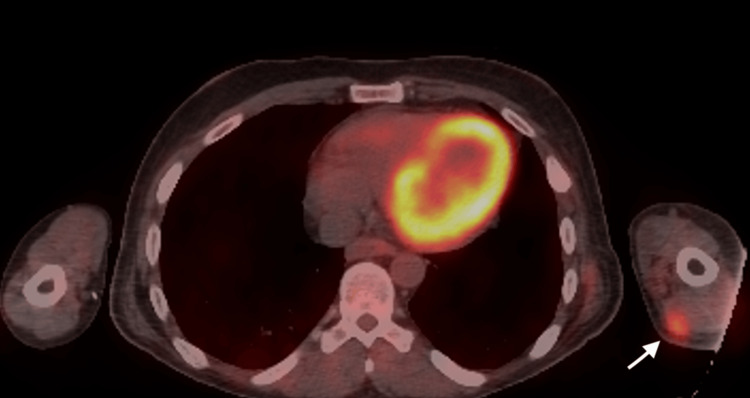
PET-CT scan (transverse view) Increased area of FDG uptake noted in the left shoulder, indicating increased metabolic activity in the region. In this case, this signifies ongoing infection in the region.

A transthoracic echocardiogram (TTE) on Day 6 showed no vegetations. However, due to persistent bacteremia, a transesophageal echocardiogram (TOE) (Figure 4) was undertaken on Day 33, revealing a vegetation on the right atrial pacemaker lead.

**Figure 4:**
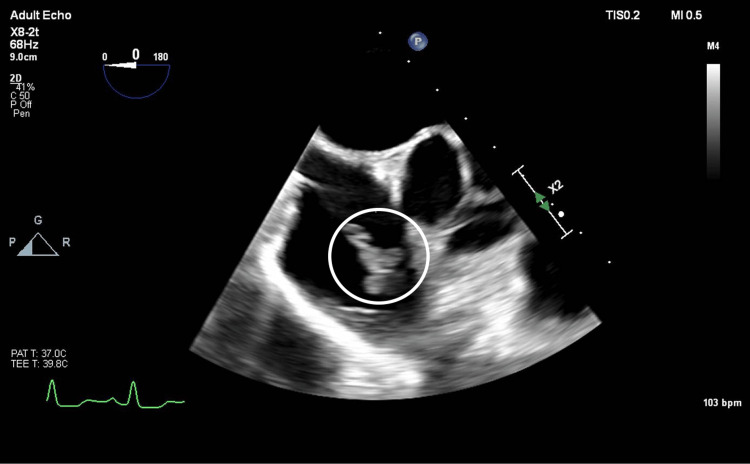
TOE The outlined area shows vegetation on the right atrial pacemaker lead. TOE, transesophageal echocardiogram

The patient was transferred to a tertiary center where he underwent complete pacemaker system extraction on Day 48; the lead tip grew coagulase-negative *Staphylococcus*. He completed four weeks of IV flucloxacillin post-extraction, with adjunct rifampicin. CRP decreased steadily, and blood cultures remained negative.

The patient later had a loop recorder inserted. Given his <1% pacing requirement, the decision was made by the cardiology multidisciplinary team to continue with the loop recorder without reimplantation of a permanent pacemaker device. He continued to improve clinically, with physiotherapy for his arm.

A summary of the timeline of key events is provided in Table 2.

**Table 2:** Summary of timeline with key events from date of admission FGM, flash glucose monitoring; TOE, transesophageal echocardiogram; TTE, transthoracic echocardiogram

Date from admission	Event
Day 0	Admission with fever, confusion, FGM-site cellulitis; CRP 421; blood cultures showing *Staphylococcus aureus*
Day 1-Day 2	Antibiotic switched from piperacillin-tazobactam to flucloxacillin
Day 2-Day 6	Persistent *S. aureus* bacteremia
Day 6	TTE showing no vegetations
Day 10	First negative blood culture result
Day 27	Shoulder washout; blood culture positive for *S. aureus*
Day 33	TOE showed pacemaker lead vegetation
Day 48	Pacemaker system extraction; lead tip culture positive
Day 52-Day 76	IV flucloxacillin with adjunctive rifampicin

## Discussion

FGM systems are widely used in patients with type 1 diabetes and are generally safe. Most reported complications are limited to mild skin irritation or local inflammation. However, insertion-site infections, although uncommon, can become clinically significant, particularly in individuals with poor glycemic control. Hyperglycemia is known to impair immune function and increase susceptibility to bacterial infection. In this case, the ulcerated and purulent FGM insertion site likely acted as the portal of entry for *S. aureus*, leading to persistent bacteremia.

Persistent *S. aureus* bacteremia is associated with a high risk of deep-seated infection and endovascular involvement. In patients with cardiac implantable electronic devices, ongoing positive blood cultures should raise suspicion for device-related infective endocarditis. Although the initial TTE was normal, the persistence of bacteremia appropriately prompted TOE, which demonstrated pacemaker lead vegetation. This highlights the limited sensitivity of transthoracic imaging in detecting lead-associated infection and supports the recommendation for TOE when clinical suspicion remains high [8].

This case also illustrates metastatic spread of infection, with hematogenous seeding of the glenohumeral joint resulting in septic arthritis and deep soft tissue abscess formation.* S. aureus *is a common cause of septic arthritis in the setting of bacteremia, and early surgical source control combined with targeted IV antibiotics was essential in achieving microbiological clearance.

Complete device extraction is recommended in confirmed cardiac device infection, as retention of infected hardware is associated with relapse and poorer outcomes. In this patient, pacemaker system removal resulted in sustained clearance of infection, and reimplantation was not required due to minimal pacing dependency.

While cutaneous complications of glucose monitoring systems have been described in the literature [6,7], progression to persistent *S. aureus* bacteremia with both septic arthritis and pacemaker lead endocarditis is rare. This case emphasizes that even apparently localized sensor-site infections may lead to serious systemic complications in high-risk patients. Clinicians should maintain a low threshold for blood cultures and advanced imaging when patients with diabetes and indwelling cardiac devices present with signs of infection.

## Conclusions

FGM insertion-site infections are uncommon but can act as a portal for *S. aureus*, with the potential for severe complications in high-risk patients. Persistent bacteremia should prompt investigation for deep infection and device-related infective endocarditis, particularly in individuals with cardiac implants. Early recognition, imaging, appropriate antibiotics, and timely device extraction are essential for effective management.
